# Clinical application values of neutrophil-to-lymphocyte ratio in intracranial aneurysms

**DOI:** 10.18632/aging.202445

**Published:** 2021-02-01

**Authors:** Baorui Zhang, Lin Lin, Fei Yuan, Guangrong Song, Qing Chang, Zhongxue Wu, Zhongrong Miao, Dapeng Mo, Xiaochuan Huo, Aihua Liu

**Affiliations:** 1 Beijing Neurosurgical Institute, Capital Medical University, Beijing 100070, China; 2 Department of Interventional Neuroradiology, Beijing Tiantan Hospital, Capital Medical University, Beijing 100070, China; 3 China National Clinical Research Center for Neurological Diseases, Beijing 100070, China; 4 Department of Information Center, Beijing Tiantan Hospital, Capital Medical University, Beijing 100070, China; 5 Department of Neurology, The First Affiliated Hospital of Hebei North University, Zhangjiakou 075000, China

**Keywords:** aneurysm, neutrophil-to-lymphocyte ratio, inflammation, endovascular treatment

## Abstract

Inflammation plays an important role in the pathogenesis and growth of intracranial aneurysms (IAs). We investigated the clinical value of the neutrophil-to-lymphocyte ratio (NLR) as a marker of systemic subclinical inflammation in patients with IAs.

Consecutive patients with IAs who underwent endovascular treatment (EVT) were enrolled in the study. The evaluation indicators were aneurysm size and rupture, a poor outcome at 3 to 6 months, and delayed cerebral ischemia (DCI) during hospitalization.

In total, 532 patients with IAs underwent EVT (mean age, 54.0 years; 62.4% female). Among patients with ruptured IAs, those with a higher NLR had an increased risk of a poor outcome at 3 to 6 months and DCI during hospitalization than those with a lower NLR. A higher NLR was significantly more strongly associated with the size of unruptured aneurysms and aneurysm rupture than a lower NLR. The NLR and C-reactive protein concentration showed similar predictive ability for aneurysm size and treatment prognosis. The NLR was lower at discharge than admission for patients with ruptured IAs and DCI.

An elevated NLR was significantly associated with the size of unruptured IAs, an increased risk of a poor outcome, and DCI in patients with ruptured IAs.

## INTRODUCTION

Inflammatory reactions can reportedly accelerate the formation and growth of intracranial aneurysms (IAs) and participate in repair of the aneurysm after endovascular treatment (EVT). [1–4] Moreover, inflammatory reactions can be activated after aneurysmal subarachnoid hemorrhage (SAH), leading to delayed cerebral ischemia (DCI) and adverse neurological outcomes. [5, 6] Therefore, attention has been focused on identification of inflammatory biomarkers that can reflect aneurysm growth and the therapeutic prognosis.

Various methods have been used to monitor aneurysm stability, such as wall enhancement on high-resolution vessel wall imaging, [7] morphological changes based on imaging follow-up, [8] and specific hemodynamic characteristics. [9] However, many of these techniques have low coverage and high false-positive or false-negative rates, seriously limiting their clinical applications.

The neutrophil-to-lymphocyte ratio (NLR) is a measure of the proportion of systemic neutrophils and lymphocytes. The NLR has been proposed to be a reliable composite marker and dynamic index of systemic inflammation, [10] and it reflects the immune response and combines information of innate and adaptive pathways. [11] More importantly, the NLR is convenient in practical applications because the neutrophil and lymphocyte counts are routinely measured during laboratory testing.

Previous studies have shown that an elevated NLR has prognostic significance for several conditions, including cardiovascular disease, [12–14] cancer, [15, 16] and peripheral artery disease. [17] Moreover, several studies have revealed that the NLR on admission can predict a poor prognosis of acute ischemic stroke after EVT [18, 19] and perihematomal edema growth after intracerebral hemorrhage. [20]

These findings suggest that evaluation of the NLR in patients with IAs might be particularly relevant because inflammation appears to play a critical role in aneurysm growth and prognosis after treatment. However, the clinical significance of the NLR in patients with IAs has not been thoroughly investigated.

In the present study, we sought to determine the associations of the NLR with aneurysm size and prognosis in patients with IAs after EVT, aiming to provide further reference data for cerebral aneurysm therapy.

## RESULTS

### Participants

**Figure 1:**
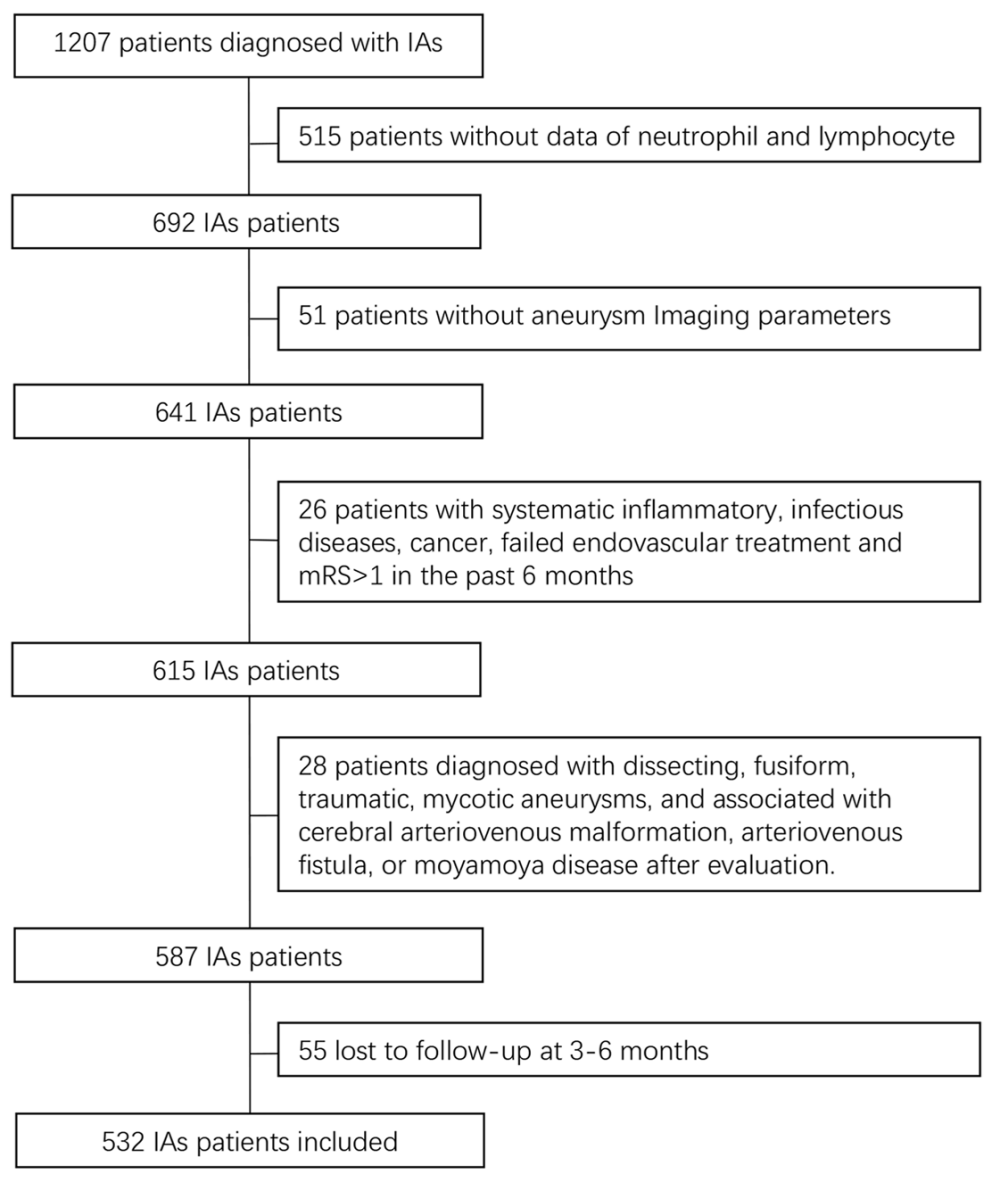
**Flow diagram of patient selection.** IAs, intracranial aneurysms.

A total of 1207 consecutive patients with IAs underwent EVT. After application of the exclusion criteria (Figure 1), 532 patients were analyzed. Among these 532 patients, the mean age was 54.0 ± 10.5 years, and 332 (62.4%) patients were women. The patients were divided into four groups as described above: the lower NLR and uIAs group comprised 363 (68.2%) patients, the higher NLR and uIAs group comprised 21 (3.9%) patients, the lower NLR and rIAs group comprised 76 (14.3%) patients, and the higher NLR and rIAs group comprised 72 (13.5%) patients. The patients’ baseline characteristics are shown in Table 1.

**Table 1:** Basic characteristics of aneurysm rupture and NLR in patients with intracranial aneurysm.

**Characteristics**	**rIAs**		**uIAs**		**p**
	**NLR<4 (n=76)**	**NLR≥4 (n=72)**	**NLR<4 (n=363)**	**NLR≥4 (n=21)**	
Age, y; mean (SD)	51.78 (10.22)	55.11 (12.57)	54.23 (10.02)	53.76 (10.56)	0.218
Women (%)	43 (56.6)	37 (51.4)	234 (64.5)	18 (85.7)	0.016
Medical history, n (%)					
Stroke, n (%)	9 (11.8)	6 (8.3)	36 (9.9)	0 (0.0)	0.461
Hypertension	36 (47.4)	45 (62.5)	173 (47.7)	12 (57.1)	0.115
Hyperlipidemia	6 (7.9)	2 (2.8)	48 (13.2)	0 (0.0)	0.012
Diabetes mellitus	4 (5.3)	3 (4.2)	38 (10.5)	4 (19.0)	0.074
Heart disease	3 (3.9)	4 (5.6)	20 (5.5)	2 (9.5)	0.707
Current smoking, n (%)	17 (22.4)	27 (37.5)	67 (18.5)	2 (9.5)	0.003
Size, mm; median (IQR)	5.23 [3.89, 7.96]	4.91 [3.52, 6.91]	5.93 [4.09, 9.79]	9.90 [6.00, 15.30]	<0.001
Location, n (%)					
Anterior circulation	12 (15.8)	3 (4.2)	55 (15.2)	1 (4.8)	0.033
Posterior circulation	64 (84.2)	69 (95.8)	308 (84.8)	20 (95.2)	
Modified fisher grade, n (%)					
0-2	59 (77.6)	46 (63.9)	NaN	NaN	NaN
>2	17 (22.4)	26 (36.1)	NaN	NaN	
Laboratory parameter					
WBC, 109/L; median (IQR)	6.46 [5.19, 7.65]	10.55 [8.35, 12.61]	5.94 [5.13, 7.07]	8.13 [7.10, 9.31]	<0.001
NEUT, 109/L; median (IQR)	3.86 [2.92, 4.70]	8.57 [6.85, 10.93]	3.45 [2.75, 4.23]	6.29 [5.71, 7.44]	<0.001
LY, 109/L; mean (SD)	2.06 (0.60)	1.14 (0.47)	2.02 (0.58)	1.15 (0.44)	<0.001
NLR, median (IQR)	1.83 [1.47, 2.46]	7.34 [5.80, 10.90]	1.77 [1.37, 2.30]	5.60 [4.52, 6.64]	<0.001
CRP, mg/L; median (IQR)	2.53 [1.90, 3.01]	9.10 [6.73, 11.70]	2.44 [1.61, 2.93]	4.27 [3.61, 5.52]	<0.001
HDL, mmol/L; mean (SD)	1.23 (0.33)	1.38 (0.36)	1.31 (0.33)	1.34 (0.38)	0.105
LDL, mmol/L; mean (SD)	2.83 [2.15, 3.42]	2.66 [2.29, 3.54]	2.84 [2.20, 3.49]	2.85 [2.06, 3.72]	0.958
TG, mmol/L; median (IQR)	1.48 [0.90, 1.93]	1.20 [0.97, 1.71]	1.29 [1.00, 1.87]	1.21 [0.76, 2.02]	0.597
Apo-A1, g/L; mean (SD)	1.35 (0.29)	1.41 (0.32)	1.48 (0.27)	1.46 (0.28)	0.008
Apo-B, g/L; mean (SD)	0.87 (0.22)	0.92 (0.24)	0.93 (0.24)	0.93 (0.24)	0.404
Body temperature, ° C; mean (SD)	37.6 (7.31)	38.1 (6.95)	36.4 (3.85)	36.7 (3.92)	0.028
Treatment Modality, n (%)					
Coiling only	50 (65.8)	61 (84.7)	118 (32.5)	4 (19.0)	NA
Stent-assisted coiling or sole stenting	26 (34.2)	10 (13.9)	235 (64.7)	17 (81.0)	
Balloon-assisted coiling and others	0 (0.0)	1 (1.4)	10 (2.8)	0 (0.0)	

rIAs, ruptured intracranial aneurysms; uIAs, unruptured intracranial aneurysms; NLR, neutrophil-lymphocyte ratio; WBC, white blood cell; NEUT, neutrophil; LY, lymphocyte; CRP, C-reactive protein; HDL, high density lipoprotein; LDL, low density lipoprotein; TG, triglyceride; ApoA-1, apolipoprotein A-I; and Apo-B, apolipoprotein B.

### Association of NLR, aneurysm size, and treatment prognosis

A total of 113 (21.2%) patients had larger aneurysms (>10 mm), and 148 (27.8%) patients had rIAs. During the 3- to 6-month follow-up, 31 (5.8%) patients had a poor outcome. Thirty-five (23.6%) of 148 patients with rIAs developed DCI. The NLR, aneurysm rupture, and risks associated with the aneurysm size and prognosis are shown in Table 2.

**Table 2:** Adjusted odds ratio of aneurysm size and prognosis by the NLR and status of aneurysm rupture.

				Model 1*		**Model 2†**	
**Characteristic**	**Groups**	**n**	**Events, n (%)**	**Adjusted OR (95% CI)**	**P Value**	**Adjusted OR (95% CI)**	**P Value**
Aneurysm size>10mm	NLR<4 with uIAs	363	86 (23.7)	Reference		Reference	
	NLR≥4 with uIAs	21	10 (47.6)	2.79 (1.14-6.85)	0.025	2.78 (1.11-6.98)	0.030
	NLR<4 with rIAs	76	12 (15.8)	Ref		Ref	
	NLR≥4 with rIAs	72	5 (6.9)	0.44 (0.14-1.33)	0.146	0.53 (0.16-1.74)	0.295
Aneurysm Rupture	NLR<4	439	76 (17.3)	Reference		Reference	
	NLR≥4	93	72 (77.4)	17.07 (9.80-29.72)	<0.001	18.46 (10.04-33.92)	<0.001
Poor outcome	NLR<4 with uIAs	363	4 (1.1)	Reference		Reference	
	NLR≥4 with uIAs	21	1 (4.8)	6.39 (0.62-66.01)	0.119	8.58 (0.37-198.94)	0.180
	NLR<4 with rIAs	76	7 (9.2)	Reference		Reference	
	NLR≥4 with rIAs	72	19 (26.4)	3.16 (1.22-8.21)	0.018	3.29 (1.07-10.15)	0.038
DCI	NLR<4 with rIAs	76	11 (14.5)	Reference		Reference	
	NLR≥4 with rIAs	72	24 (33.3)	2.85 (1.26-6.45)	0.012	2.75 (1.12-6.73)	0.027

rIAs, ruptured intracranial aneurysms; uIAs, unruptured intracranial aneurysms; NLR, neutrophil-lymphocyte ratio; and DCI, delayed cerebral ischemia; CI, confidence interval; OR, odds ratio.

*Model 1: adjusted for age and sex.

**†**Model 2:

Size: adjusted for model 1+ history of stroke, hypertension, hyperlipidemia, diabetes mellitus, heart disease, smoking status, and aneurysm location.

Aneurysm rupture: adjusted for model 1+ history of stroke, hypertension, hyperlipidemia, diabetes mellitus, heart disease, smoking status, aneurysm location, and aneurysm size.

Poor outcome: adjusted for model 1+ history of stroke, hypertension, hyperlipidemia, diabetes mellitus, heart disease, smoking status, aneurysm location, aneurysm size, treatment modality, and modified Fisher grade.

DCI: adjusted for model 1+ history of stroke, hypertension, hyperlipidemia, diabetes mellitus, heart disease, smoking status, aneurysm location, aneurysm size, treatment modality, and modified Fisher grade.

Among patients with uIAs, a higher NLR was significantly more strongly associated with a larger aneurysm size than was a lower NLR (OR, 2.78; 95% CI, 1.11–6.98; P = 0.03) after adjustment for the variables in Model 2 (history of stroke, hypertension, hyperlipidemia, diabetes mellitus, heart disease, smoking status, aneurysm size, and aneurysm location). A higher NLR was also significantly more strongly associated with aneurysm rupture than was a lower NLR (OR, 18.46; 95% CI, 10.04–33.92; P < 0.001) after adjustment for the variables in Model 2 (history of stroke, hypertension, hyperlipidemia, diabetes mellitus, heart disease, smoking status, aneurysm size, aneurysm location, and aneurysm size).

Among patients with rIAs, those with a higher NLR had a 3.29-fold higher risk of a poor outcome at 3 to 6 months than those with a lower NLR after adjustment for the variables in Model 2 (history of stroke, hypertension, hyperlipidemia, diabetes mellitus, heart disease, smoking status, aneurysm size, aneurysm location, treatment modality, and modified Fisher grade). Patients with a higher NLR also had a 2.75-fold higher risk of DCI than patients with a lower NLR after adjustment for the variables in Model 2 (history of stroke, hypertension, hyperlipidemia, diabetes mellitus, heart disease, smoking status, aneurysm size, aneurysm location, treatment modality, and modified Fisher grade).

**Figure 2:**
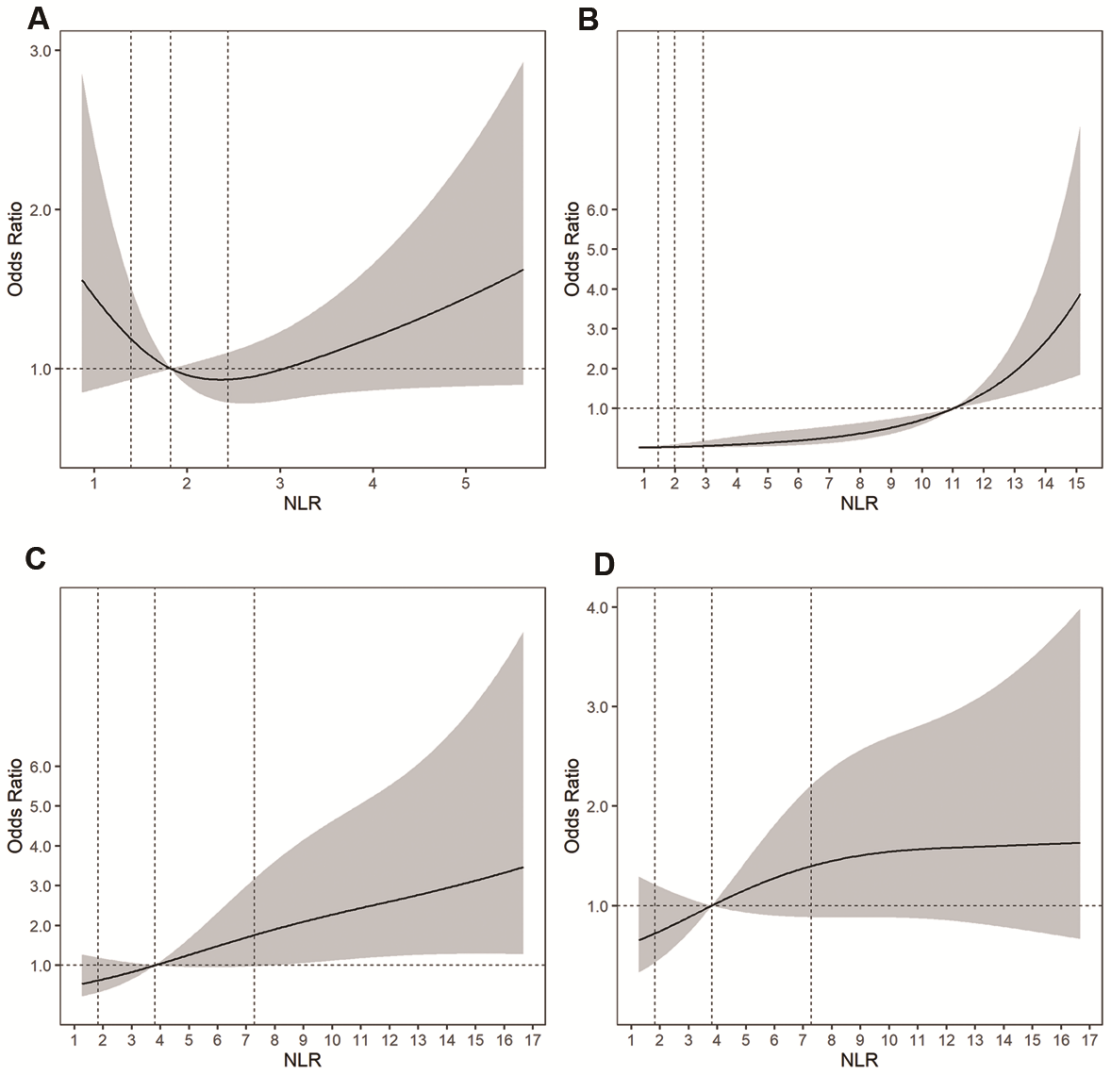
Adjusted ORs of (**A**) aneurysm size for uIAs, (**B**) aneurysm rupture, (**C**) a poor outcome at 3 to 6 months for rIAs, and (**D**) DCI for rIAs according to the NLR. The solid line indicates the adjusted OR and the shadow the 95% confidence interval bands. The reference is the 50th percentile of the NLR for aneurysm size, DCI, and a poor outcome, and NLR = 11.0 for aneurysm rupture. The other vertical dashed lines indicate the first, second, and third quartiles of the NLR. The data were fitted using a logistic regression model of restricted cubic splines with three knots for the NLR, adjusting for potential covariates as Model 2 in Table 2. The lowest 5% and highest 5% of participants are not shown in the figures. ORs, odds ratios; rIAs, ruptured intracranial aneurysms; uIAs, unruptured intracranial aneurysms; NLR, neutrophil-to-lymphocyte ratio; DCI, delayed cerebral ischemia.

Using a logistic regression model with restricted cubic splines, we found a U-shaped association between the NLR and the risk of a larger aneurysm size and positive associations between the NLR and the risk of aneurysm rupture, DCI during hospitalization, and a poor outcome at 3 to 6 months (Figure 2).

### Correlation of NLR and CRP, and comparison of these parameters for prediction of aneurysm size and treatment prognosis

**Figure 3:**
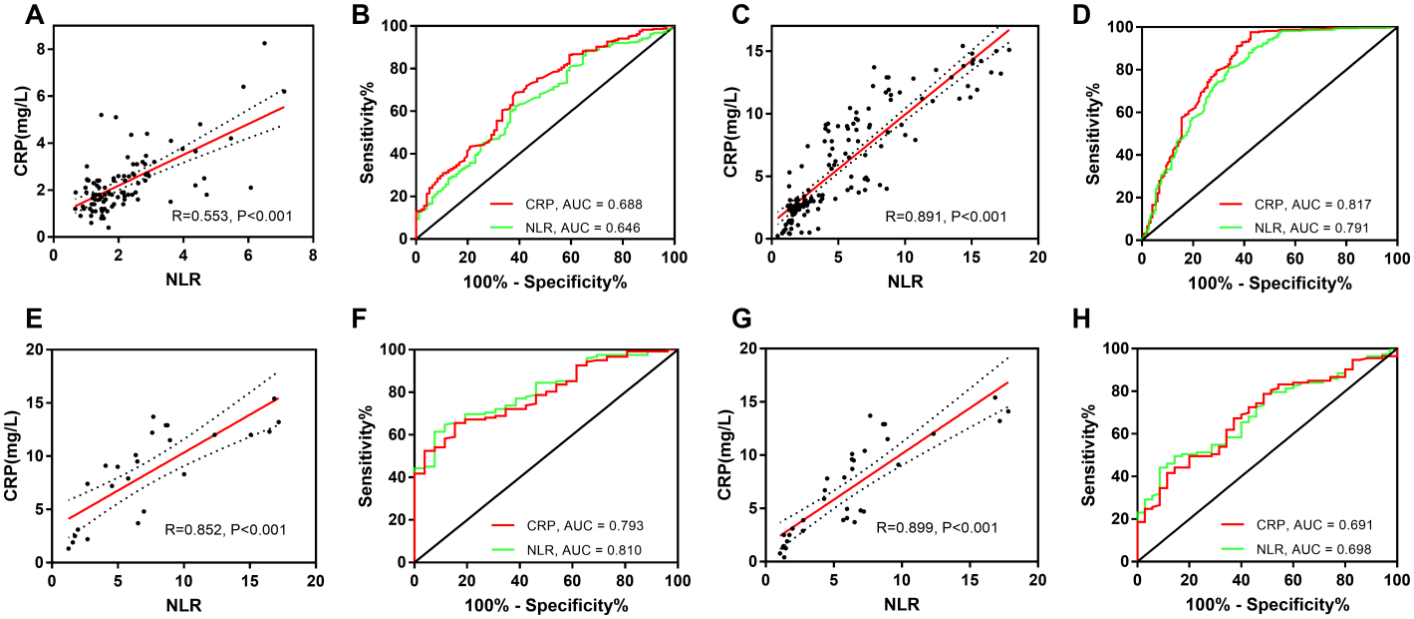
**Correlation of NLR and CRP, and comparison of these parameters for prediction of aneurysm size and treatment prognosis.** Correlation of the NLR and CRP concentration for (**A**) uIA, (**C**) rIAs, (**E**) a poor outcome, and (**G**) DCI. Comparison of the NLR and CRP concentration for prediction of (**B**) uIA size, (**D**) aneurysm rupture, (**F**) a poor outcome, and (**H**) DCI. NLR, neutrophil-to-lymphocyte ratio; CRP, C-reactive protein; uIA, unruptured intracranial aneurysms; rIAs, ruptured intracranial aneurysms; DCI, delayed cerebral ischemia.

The NLR and C-reactive protein concentration were significantly correlated in patients with larger uIAs (r = 0.553, P < 0.001), rIAs (r = 0.891, P < 0.001), poor outcomes (r = 0.852, P < 0.001), and DCI (r = 0.899, P < 0.001). The areas under the receiver operating characteristic curve of the NLR and C-reactive protein concentration for the prediction of uIA size, rIAs, poor outcomes, and DCI were as follows: [0.646 (95% CI, 0.583–0.709) vs. 0.688 (95% CI, 0.627–0.749), P = 0.066], [0.791 (95% CI, 0.744–0.838) vs. 0.817 (95% CI, 0.772–0.863), P = 0.019], [0.810 (95% CI, 0.733–0.888) vs. 0.793 (95% CI, 0.713–0.874), P = 0.252], and [0.698 (95% CI, 0.608–0.789) vs. 0.691 (95% CI, 0.598–0.785), P = 0.735], respectively (Figure 3, Table 3).

**Table 3:** Areas under ROC of NLR and CRP concentration to predict the aneurysm size and treatment prognosis.

	**Items**	**Aneurysm size>10mm**	**Aneurysm rupture**	**Poor outcome**	**DCI**
C-statistics	CRP	0.688	0.817	0.793	0.691
	NLR	0.646	0.791	0.810	0.698
P value of paired comparisons		0.066	0.019	0.252	0.735

ROC, receiver operating characteristic curve; NLR, neutrophil-to-lymphocyte ratio; CRP, C-reactive protein; DCI, delayed cerebral ischemia.

### Comparison of overall white blood cell count, neutrophil count, lymphocyte count, and NLR at admission and discharge in patients with rIAs and DCI

Among patients with rIAs, we found that the overall white blood cell count was lower at discharge than at admission [7.57 (IQR, 6.25–9.53) vs. 8.97 (IQR, 6.88–11.73), P < 0.001], that the neutrophil count was reduced [5.29 (IQR, 4.04–6.94) vs. 7.36 (IQR, 4.70–9.97), P < 0.001], and that the lymphocyte count was increased [1.54 (IQR, 1.19–2.01) vs. 1.33 (IQR, 0.94–1.76), P < 0.001], resulting in a significantly lower NLR at discharge than at admission [3.10 (IQR, 2.22–5.36) vs. 5.92 (IQR, 2.71–8.77), P < 0.001]. Among patients with DCI, we found that the overall white blood cell count was lower at discharge than at admission [8.45 (IQR, 6.11–10.43) vs. 10.27 (IQR, 8.33–12.28), P = 0.006], that the neutrophil count was reduced [5.93 (IQR, 4.38–8.35) vs. 8.34 (IQR, 6.52–10.06), P = 0.003], and that the lymphocyte count was increased [1.55 (IQR, 1.12–2.00) vs. 1.31 (IQR, 1.06–1.70), P = 0.066], resulting in a significantly lower NLR at discharge than at admission [3.50 (IQR, 2.59–6.08) vs. 6.36 (IQR, 4.35–8.75), P = 0.027] (Table 4).

**Table 4:** Comparison of overall white blood cell count, neutrophil count, lymphocyte count and NLR at admission and discharge.

**Items**		**At admission**	**At discharge**	**P**
Ruptured aneurysms	Overall WBC	8.97 [6.88, 11.73]	7.57 [6.25, 9.53]	<0.001
	NEUT	7.36 [4.70, 9.97]	5.29 [4.04, 6.94]	<0.001
	LY	1.33 [0.94, 1.76]	1.54 [1.19, 2.01]	<0.001
	NLR	5.92 [2.71, 8.77]	3.10 [2.22, 5.36]	<0.001
DCI	Overall WBC	10.27 [8.33, 12.28]	8.45 [6.11, 10.43]	0.006
	NEUT	8.34 [6.52, 10.06]	5.93 [4.38, 8.35]	0.003
	LY	1.31 [1.06, 1.70]	1.55 [1.12, 2.00]	0.066
	NLR	6.36 [4.35, 8.75]	3.50 [2.59, 6.08]	0.027

WBC, white blood cell; NLR, neutrophil-lymphocyte ratio; NEUT, neutrophil; LY, lymphocyte; DCI, delayed cerebral ischemia.

## DISCUSSION

Among patients with rIAs who underwent EVT, those with a higher NLR had a 3.29-fold higher risk of a poor outcome at 3 to 6 months and a 2.75-fold higher risk of DCI during hospitalization than those with a lower NLR. Among patients with uIAs, a higher NLR was significantly more closely associated with aneurysm size (2.78-fold) and aneurysm rupture (18.46-fold) than was a lower NLR. The NLR and C-reactive protein concentration showed similar predictive ability for aneurysm size and treatment prognosis. The NLR was lower at discharge than at admission for patients with rIAs and DCI.

The prediction of aneurysm growth is currently based on follow-up images, which are used to judge whether the aneurysm is growing. Wall enhancement on high-resolution magnetic resonance imaging can reflect the presence of inflammatory changes in the aneurysm wall associated with aneurysm growth. [21, 22] Pathologically, aneurysm growth is mainly related to impaired function of vascular smooth muscle cells, which can induce destruction of the internal elastic lamina and cause dysregulation of collagen synthesis and remodeling of the extracellular matrix in human cerebral aneurysmal walls. [23, 24] At the same time, neutrophils, lymphocytes, and various cytokines and inflammatory mediators are involved in phenotypic modulation of vascular smooth muscle cells. [25, 26] In this study, we found that the NLR was strongly associated with the size of uIAs, indicating that neutrophils and lymphocytes may participate in the growth of aneurysms to some extent. The concept that neutrophils are involved in the development of aneurysms has been demonstrated from various perspectives; for example, the level of neutrophil RNA was higher in patients with IAs than in aneurysm-free controls, [27] and neutrophil recruitment was found to aggravate the development of abdominal aortic aneurysms. [28] In this study, we found a U-shaped association between the NLR and risk of aneurysm growth. Thus, the innate immunity provided by neutrophils and the adaptive immunity provided by lymphocytes may orchestrate a local inflammatory response in the aneurysm wall, inducing aneurysm growth. [11, 29–31] However, two points must be noted. First, the inflammation reflected by the NLR may be manifested by the aneurysm local mass effect and aneurysm microhemorrhage. Second, our study showed that a higher NLR was more strongly associated with the growth of uIAs than rIAs, which may be related to the morphologic changes that occur in the aneurysm after rupture. In summary, although the NLR may be useful for clinically judging whether an aneurysm will grow, this still needs to be verified by prospective cohort studies.

We further discovered that a higher NLR was combined with a poor outcome after EVT for rIAs. The same conclusion has also been found in other studies; however, many of them did not indicate the effect of the treatment strategy on the outcome. [32, 33] One study indicated that patients with rIAs and an elevated NLR on admission had higher in-hospital mortality, but insufficient statistical efforts were undertaken to adjust for obvious confounders. [34] Another study showed that the NLR can independently predict the functional outcome after aneurysm rupture; however, the authors did not adjust for age in the multivariable regression model. [32] Our study adds evidence for an association between the NLR and poor outcomes of aneurysmal SAH after EVT. This may be of significance to help evaluate the outcome of aneurysmal SAH treated with EVT in clinical practice.

In this study, an elevated NLR was associated with aneurysm rupture, which we believe is mainly related to activation of the downstream inflammatory cascade associated with aneurysmal SAH. [5] However, we cannot rule out the presence of a higher NLR before aneurysm rupture. Previous studies have shown that larger aneurysms are more prone to rupture, [35, 36] and we found that an elevated NLR was associated with large aneurysms; therefore, an elevated NLR may be a manifestation before aneurysm rupture. Moreover, some studies have shown that a higher NLR is associated with rupture of aortic arch aneurysms, which is also consistent with our findings. [14, 37] A recent study also showed that the NLR is associated with rupture of cerebral aneurysms. [38] This further confirms our conjecture. Because the blood samples were collected after aneurysm rupture in the present study, the relationship between the NLR and aneurysm rupture requires verification in further prospective observational studies.

Several studies have shown that the NLR can independently predict DCI after aneurysmal SAH; however, most of these studies did not analyze the effect of EVT or surgical clipping on DCI, [39, 32] thus limiting the strength of the study conclusions in patients undergoing EVT. To exclude the effect of the treatment strategy on the results, we enrolled all patients who underwent EVT. A previous study indicated that ethnicity affected the admission NLR in patients with aneurysmal SAH. [39] In the present study, however, all patients were of Han ethnicity. This allowed us to avoid the ethnicity effect, but our results also show that the ability of the NLR to predict DCI after aneurysmal SAH applies equally to patients of Han ethnicity.

Several limitations should be considered when interpreting our findings. First, this was a single-center, retrospective study, which inevitably produced systematic bias. Second, other inflammatory markers such as interleukin 6 were not evaluated in this study. Third, the observational study design did not allow us to establish a cause–effect relationship. Fourth, because of the limitations of the retrospective data, we did not evaluate the dynamic NLR. Further studies are needed to investigate the effects of dynamic changes in the NLR on the outcomes of patients with aneurysms. Finally, more extensive research is needed to clarify the detailed mechanisms through which hematological markers influence the prognosis of patients with aneurysms after EVT.

## CONCLUSIONS

An elevated serum NLR is significantly associated with the size of uIAs, an increased risk of a poor outcome at 3 to 6 months, and DCI in patients with rIAs during hospitalization. These results indicate that the serum NLR can be used to monitor the growth of aneurysms and assess the prognosis. Finally, we recognize that this was a single-center retrospective study and therefore requires external verification.

## MATERIALS AND METHODS

### Study design and ethics

We performed a retrospective analysis of a prospectively collected database of consecutive patients with IAs who had undergone EVT in our neuro-interventional department from January 2013 to December 2016. The diagnosis of IAs was confirmed by digital subtraction angiography. The exclusion criteria were the absence of laboratory blood test data within 24 hours after admission; failed EVT of aneurysms; systematic inflammatory disease, infectious disease, or cancer; diagnosis of fusiform, traumatic, or mycotic IAs; aneurysms accompanied by arteriovenous malformations, arteriovenous fistulas, or moyamoya disease; a modified Rankin scale (mRS) score of >1 in the past 6 months; and a follow-up duration of <3 months.

This study was approved by the institutional review board of Beijing Tiantan Hospital and conformed to the tenets of the Declaration of Helsinki. Each participant or his/her representative provided informed consent.

### Data collection

Demographic information was collected through face-to-face interviews by a trained neurologist. Data regarding vascular risk factors, such as a history of stroke, hypertension, hyperlipidemia, diabetes mellitus, heart disease, and smoking, were also collected.

Venous blood samples were collected within 24 hours after admission and placed in K2-EDTA anticoagulation tubes. A complete blood count, including white blood cell, the neutrophil and lymphocyte counts, was performed using an autoanalyzer (Mindray Hematology Analyzer BC-6900 series; Mindray Corporation, Shenzhen, China). The concentrations of C-reactive protein, total cholesterol, triglycerides, high-density lipoprotein cholesterol, low-density lipoprotein cholesterol, apolipoprotein A1, and apolipoprotein B were measured using an autoanalyzer (Hitachi Automatic Biochemical Analyzer 7600-020 series; Hitachi Corporation, Tokyo, Japan).

The NLR was defined as the quotient of the baseline absolute peripheral neutrophil and lymphocyte counts. The patients were categorized into four groups according to the NLR (NLR of <4 and ≥4 based on a previously published study) [40] and aneurysm rupture: a lower NLR and unruptured IAs (uIAs), a higher NLR and uIAs, a lower NLR and ruptured IAs (rIAs), and a higher NLR and rIAs.

### Image collection and analysis

The diagnosis of rIAs was established by admission computed tomography (CT), which also was utilized to evaluate the SAH severity as reflected by the modified Fisher grade. The aneurysm size was defined as the maximum diameter of the IAs measured on two-dimensional imaging and three-dimensional digital subtraction angiography using the Siemens Artis Zee System (Siemens, Erlangen, Germany). Anterior circulation aneurysms were those located in the anterior cerebral artery, anterior communicating artery, middle cerebral artery, internal carotid artery, and posterior communicating artery, whereas posterior circulation aneurysms were located in the basilar artery, posterior cerebral artery, and intracranial segment of the vertebral artery. All images were read by two neurointerventionists (B.Z. and X.H.) who were blinded to each other’s interpretations and the patients’ clinical information. Disagreements were resolved by a third reader (A.L.).

### Treatment strategy

Treatment modalities and indications were based on the patients’ conditions and determined by a neurovascular team through an interdisciplinary decision-making process. The preferences of the patients and their families also served as a reference. EVT was considered the primary treatment for uIAs. [41] Aneurysm occlusion was performed as soon as possible but was postponed in patients with a poor clinical condition. For rIAs, the treatment modality (surgical clipping vs. EVT) was determined by the individual patient’s condition, size and location of the aneurysm, and amount of bleeding. [42] EVT mainly involved coiling, stent-assisted coiling, and balloon-assisted coiling; the final treatment decision was made by the operator. CT was routinely performed on day 1, 7, and 14 after the procedure or earlier if neurologic worsening occurred. In patients with rIAs, an external ventricular drain or, when possible, a lumbar catheter was placed for cerebrospinal fluid drainage in case of hydrocephalus or if high intracranial pressure was suspected.

### Outcome assessment

Clinical follow-up supplemented by telephone interviews was performed 3 to 6 months after EVT and annually thereafter. The neurological status was measured using the mRS, and a poor outcome was defined as an mRS score of >2. [43]

DCI was assessed during hospitalization and defined as (1) the occurrence of focal neurologic impairment, a 2-point decrease in the Glasgow coma scale score, or the development of focal neurological impairment (including neglect, hemiparesis, aphasia, hemianopia, or apraxia) that could not be attributed to other causes or (2) a new low-density area that had not been seen on a previous CT scan and was not attributable to other causes (such as treatment) or a low-density shadow after absorption of a hematoma. [44]

### Statistical analysis

The baseline variables were compared between groups using the χ^2^ test or Fisher’s exact test for categorical variables and one-way analysis of variance or the Kruskal–Wallis test for continuous variables with a normal or skewed distribution, respectively. Continuous variables are expressed as mean ± standard deviation or median with interquartile range (IQR). Categorical data are presented as proportions.

The associations of each NLR category with the aneurysm size and prognosis were estimated using a multivariable logistic regression model. Adjusted odds ratios (ORs) and their 95% confidence intervals (CIs) were calculated. Two models were used in the multivariable regression analyses. In the first model, we only adjusted for age and sex. In the second model, we included all potential confounding variables.

We evaluated the pattern and magnitude of the associations between the NLR and aneurysm size and prognosis using a logistic regression model with restricted cubic splines with three knots for the NLR, adjusting for all potential covariates (Model 2).

We also assessed the correlation between the NLR and C-reactive protein concentration using Spearman’s rank correlation coefficient. We compared the ability of the NLR and C-reactive protein concentration to predict the aneurysm size and treatment prognosis after adjusting for all potential covariates (Model 2). The areas under the receiver operating characteristic curves (c-statistics) were compared to evaluate the predictive ability of the NLR and C-reactive protein concentration using the DeLong method. [45]

The Wilcoxon signed-rank test was used to assess changes in the overall white blood cell count, neutrophil count, lymphocyte count, and NLR at admission and discharge.

Two-sided P values of <0.05 were considered statistically significant. All statistical analyses were performed using R software version 3.6.3 (R Foundation for Statistical Computing, Vienna, Austria) and MedCalc Statistical Software version 19.0.7 (MedCalc Software Bvba, Ostend, Belgium; https://www.medcalc.org; 2019).
